# Supplementary material for: Respiratory syncytial virus, human metapneumovirus, and influenza virus infection in Bangkok, 2016-2017
Source: PeerJ. 2019 Apr 11;7:e6748. doi: 10.7717/peerj.6748 (PMC6462397; doi:10.7717/peerj.6748)
Supplement: Table S1 [file peerj-07-6748-s001.docx]

**Table S1:**

**Reference strain of RSV for construction partial G gene phylogenetic tree.**

| **Type** | **Strain** | **GenBank**  **accession number** | **Genotype** |
| --- | --- | --- | --- |
| RSV-A | A2 (wildtype) | M74568 | GA1 |
|  | MO55 | AF233915 | GA2 |
|  | MO13 | AF233911 | GA3 |
|  | CH09 | AF065254 | GA4 |
|  | MO01 | AF233909 | GA5 |
|  | NY20 | AF233918 | GA6 |
|  | MO02 | AF233910 | GA7 |
|  | SA98V603 | AF348807 | SAA1 |
|  | NG-016-04 | AB470478 | NA1 |
|  | NG-082-05 | AB470479 | NA2 |
|  | BJ/28170 | KC297260 | NA3 |
|  | BJ/36578 | KC297374 | NA4 |
|  | ON67-1210A | JN257693 | ON1 |
| RSV-B | B1 (wildtype) | AF013254 | GB1 |
|  | CH93-9b | AF065251 | GB2 |
|  | MO35 | AF233929 | GB3 |
|  | NY01 | AF233931 | GB4 |
|  | SA0025 | AF348825 | SAB1 |
|  | SA99V800 | AF348821 | SAB2 |
|  | SA99V429 | AF348813 | SAB3 |
|  | Cam2009-1013 | JN119979 | SAB4 |
|  | Mon/7/01 | AY488804 | URU1 |
|  | Mon/15/90 | AY333361 | URU2 |
|  | CU2010/5 | KC342336 | THB |
|  | BE/13417/99 | AY751131 | BA1 |
|  | BA/1161/02 | DQ227377 | BA2 |
|  | BA/1004/02 | DQ227376 | BA3 |
|  | BA/493/04 | DQ227407 | BA4 |
|  | NG-006-03 | AB175820 | BA5 |
|  | BE/13159/02 | AY751117 | BA6 |
|  | NG-068-05 | HM459864 | BA7 |
|  | NG-228-06 | HM459871 | BA8 |
|  | NG-119-07 | HM459878 | BA9 |
|  | NG-042-07 | HM459884 | BA10 |
|  | BJ/F9743 | KC297486 | BA-C |
