# Supplementary material for: Respiratory syncytial virus, human metapneumovirus, and influenza virus infection in Bangkok, 2016-2017
Source: PeerJ. 2019 Apr 11;7:e6748. doi: 10.7717/peerj.6748 (PMC6462397; doi:10.7717/peerj.6748)
Supplement: Table S2 [file peerj-07-6748-s002.docx]

**Table S2:**

**Reference strain of hMPV for construction F gene phylogenetic tree.**

| Type | Strain | GenBank  accession number | Genotype |
| --- | --- | --- | --- |
| hMPV-A | CAN99-81 | AY574224 | A1 |
|  | NL/17/00 | AY304360 | A2 |
|  | CAN97-83 | AY145296 | A2 |
| hMPV-B | NL/1/99 | AY304361 | B1 |
|  | CAN97-82 | AY145295 | B1 |
|  | CAN98-75 | AY145289 | B2 |
|  | NL/1/94 | AY304362 | B2 |
