# Supplementary material for: Respiratory syncytial virus, human metapneumovirus, and influenza virus infection in Bangkok, 2016-2017
Source: PeerJ. 2019 Apr 11;7:e6748. doi: 10.7717/peerj.6748 (PMC6462397; doi:10.7717/peerj.6748)
Supplement: Table S3 [file peerj-07-6748-s003.docx]

**S3 Table:**

The correlation of virus prevalence and age was assessed using univariate analyses.

|  | **Age** | ≤2 | 3-5 | 6-12 | 13-18 | 19-30 | 31-60 | >60 |
| --- | --- | --- | --- | --- | --- | --- | --- | --- |
| RSV | ≤2 |  |  |  |  |  |  |  |
|  | 3-5 | **0.000** |  |  |  |  |  |  |
|  | 6-12 | **0.000** | **0.000** |  |  |  |  |  |
|  | 13-18 | **0.000** | **0.000** | 0.069 |  |  |  |  |
|  | 19-30 | **0.000** | **0.000** | **0.036** | 0.699 |  |  |  |
|  | 31-60 | **0.000** | **0.000** | 0.148 | 0.303 | 0.331 |  |  |
|  | >60 | **0.000** | **0.000** | 0.763 | 0.073 | 0.055 | 0.185 |  |
| hMPV | ≤2 |  |  |  |  |  |  |  |
|  | 3-5 | **0.000** |  |  |  |  |  |  |
|  | 6-12 | **0.000** | **0.000** |  |  |  |  |  |
|  | 13-18 | **0.026** | **0.018** | 0.417 |  |  |  |  |
|  | 19-30 | **0.000** | **0.000** | 0.764 | 0.282 |  |  |  |
|  | 31-60 | **0.000** | **0.000** | 0.409 | 0.072 | 0.214 |  |  |
|  | >60 | 0.156 | 0.119 | 0.049 | 0.436 | 0.025 | 0.110 |  |
| Influenza | ≤2 |  |  |  |  |  |  |  |
| virus | 3-5 | **0.000** |  |  |  |  |  |  |
|  | 6-12 | **0.000** | **0.000** |  |  |  |  |  |
|  | 13-18 | **0.000** | **0.000** | 0.192 |  |  |  |  |
|  | 19-30 | **0.000** | **0.000** | **0.001** | **0.000** |  |  |  |
|  | 31-60 | **0.000** | **0.000** | 1.000 | 0.169 | **0.000** |  |  |
|  | >60 | **0.000** | **0.000** | 0.898 | 0.334 | **0.012** | 0.952 |  |

P-values were calculated using the Chi-squared test or Fisher’s exact test, where cell counts below 5 were used. P-values less than 0.05 are indicated in bold.
